# An autoregressive logistic model to predict the reciprocal effects of oviductal fluid components on *in vitro* spermophagy by neutrophils in cattle

**DOI:** 10.1038/s41598-017-04841-z

**Published:** 2017-06-30

**Authors:** Rasoul Kowsar, Behrooz Keshtegar, Mohamed. A. Marey, Akio Miyamoto

**Affiliations:** 1 Department of Animal Sciences, College of Agriculture, Isfahan University of Technology, Isfahan, 84156–83111 Iran; 2 Department of Civil Engineering, Faculty of Engineering, University of Zabol, P.B. 9861335-856 Zabol, Iran; 3 Faculty of Veterinary Medicine, Damanhur University, Behera, Egypt; 4 Graduate School of Animal and Food Hygiene, Obihiro University of Agriculture and Veterinary Medicine, Obihiro, Hokkaido 080-8555 Japan

## Abstract

After intercourse/insemination, large numbers of sperm are deposited in the female reproductive tract (FRT), triggering a massive recruitment of neutrophils (PMNs) into the FRT, possibly to eliminate excessive sperm *via* phagocytosis. Some bovine oviductal fluid components (BOFCs) have been shown to regulate *in vitro* sperm phagocytosis (spermophagy) by PMNs. The modeling approach-based logistic regression (LR) and autoregressive logistic regression (ALR) can be used to predict the behavior of complex biological systems. We, first, compared the LR and ALR models using *in vitro* data to find which of them provides a better prediction of *in vitro* spermophagy in bovine. Then, the best model was used to identify and classify the reciprocal effects of BOFCs in regulating spermophagy. The ALR model was calibrated using an iterative procedure with a dynamical search direction. The superoxide production data were used to illustrate the accuracy in validating logit model-based ALR and LR. The ALR model was more accurate than the LR model. Based on *in vitro* data, the ALR predicted that the regulation of spermophagy by PMNs in bovine oviduct is more sensitive to alpha-1 acid glycoprotein (AGP), PGE2, bovine serum albumin (BSA), and to the combination of AGP or BSA with other BOFCs.

## Introduction

In addition to sperm transportation and distribution, the extension of sperm elimination and sperm biology within the female reproductive tract (FRT) play a critical role in fertility or sub-fertility and eventually in reproductive success in mammals i.e. bovines^1, 2^. Indeed, after intercourse or insemination, millions or billions of sperm are deposited either in the anterior vagina (primates, ruminants), the cervix (most mammalian species) or in the uterus (pig, horse)^3–5^. However, a limited number (hundreds to thousands) reaches the bovine oviduct as the fertilisation site^6, 7^. Actually, it has been shown that polymorphonuclear neutrophils (PMNs) exist in the bovine oviductal flushes^8^ and massive recruitment of PMNs into the FRT occurs after insemination^9–11^. Since spermatozoa are able to activate neutrophils^12^, it seems that sperm can be eliminated from the FRT through phagocytosis by PMNs. It has been shown that in humans^13^, cats^14^, and mice^15^ the remained/post-fertilization sperm cells in the oviduct are phagocytosed by isthmic epithelial cells and leukocytes^15, 16^. Indeed, post-capacitated sperm cells which lost their fertilizability have been shown to induce migration of leukocytes into the FRT, undergo phagocytosis and, thereby, are eliminated from the FRT^16^. This implies that phagocytosis of sperm by epithelial cells or PMNs, as a part of normal physiology of the female body, acts not to intervene in the fecundity. This also suggests that there may be some cross talks between fertilizing sperm and phagocytosing cells such that the dead sperm or remained sperm after fertilization may be losing these cross-talks. For example, in comparison with live sperm cells, dead or abnormal sperm cells failed to induce prostaglandin (PG) system including *PGES* and *COX-2* expression and PGE2 secretion by BOECs^17^. However, little is done to reveal what factors in the FRT contribute in establishing such cross-talks. Our group has tried to reveal the possible role of physiological factors i.e. sperm or hormones, in establishing such cross-talks between bovine oviductal fluid components (BOFCs), sperm, and phagocytosing cells. Of importance, we have reported that binding of live sperm to bovine oviductal epithelial cells induces PGE2 secretion which, in turn, reduces bovine spermophagy by PMN^8, 17^ and also skews the local innate immunity toward an anti-inflammatory microenvironment^18^. In addition, spermophagy has been also shown to be regulated by bovine oviduct epithelial culture medium and by individual BOFCs like endothelin-1 (EDN-1), PGE2, angiotensin II (ANGII), and alpha 1-acid glycoprotein (AGP)^8, 19–21^.

It is of importance to accurately identify and classify the key and most influencing BOFCs involving in spermophagy by PMNs using the present experimental data. Moreover, the prediction of interactions among BOFCs can be used not only to predict and design future experiments without doing the additional expensive tests but also to gain further understanding and prediction of possible interaction between factors involving in reproductive physiology of bovine oviduct. No previous studies have applied computational modeling to confirm or classify the present *in vitro* data or to predict the reciprocal effects of BOFCs in regulating spermophagy by PMNs. Various models and methods, such as logistic regression^22–25^, support vector machine (SVM)^25^, and tree model^26^ can be used to detect complex patterns within data sets. Dose-response models include a range of statistical models such as nonlinear regression, generalized (non) linear regression, and parametric survival analysis^27^. Also, the experiments are showing the dose-dependent regulation of spermophagy by BOFCs^8, 19–21^; thus, the logistic function can be used to predict spermophagy on the basis of dose-response data^27^. Therefore, we hypothesized that an autoregressive logistic regression (ALR) model may prove to be a more powerful method for identifying and classifying the BOFCs, alone or in combination with each other, in regulating spermophagy by PMNs in cattle. A multi- study analysis was designed to construct an effective predictive model for spermophagy with high sensitivity, specificity, positive predictive value (PPV), and negative predictive value (NPV).

In this study, the predictive performance of the parametric model (LR) was compared with that of a model constructed using the ALR model. We, therefore, examined which model, the LR model or the ALR model, predicts *in vitro* spermophagy more efficiently and also tried to identify and classify that which of BOFCs, alone or in combination together, shows greater impact on *in vitro* spermophagy by PMNs in bovines.

## Results

This section consists of three applications as: 1) comparison of the logit regression (LR) and autoregressive logit regression (ALR) using several comparative error statistics to establish the better model for an accurate prediction and classification; 2) confirmation and classification of the effect of each of BOFCs on reducing spermophagy using the best logit model observed in application 1; and 3) analysis of the reciprocal effects of BOFCs in regulating spermophagy by PMNs in bovines using ALR model.

### Comparative prediction of the LR and ALR models for *in vitro* spermophagy by PMNs in cattle

As shown in Table 1, the value of the penalty coefficient set to 1.75 results in the reduction of errors and Akaike Information criteria, *AIC*, and the best *EF* level in the ALR model. Thus, the best logistic function among the various logit models are obtained based on the penalty factor λ = 1.75. The ALR model transformation relation is given as:

1 $$\begin{array}{rcl}f({\boldsymbol{\beta }}X) & = & -0.338+3.81\times {10}^{-4}BS+4.76\times {10}^{-7}LH+7.66\times {10}^{-7}BO\\  &  & -\,1.33\times {10}^{-4}AN+4.39\times {10}^{-6}P2+9.62\times {10}^{-6}AG+3.09\times {10}^{-11}ED\end{array}$$

**Table 1:** Statistical errors to compare the logit models in different penalty coefficients i.e 0.25 ≤ λ ≤ 2.

*λ*	EF	d	RMSE	MAE	AIC
0.25	0.5871	0.8504	0.0804	0.0625	−130.179
0.5	0.5864	0.8613	0.0805	0.0627	−130.132
0.75	0.5823	0.8583	0.0809	0.0630	−129.842
1	0.5890	0.8607	0.0803	0.0624	−130.313
1.25	0.5893	0.8632	0.0802	0.0617	−130.337
1.5	0.5833	**0.8635**	0.0808	0.0614	−129.912
1.75	**0.5910** ^*^	0.8616	**0.0801**	**0.0612**	**−130.457**
2	0.5732	0.8581	0.0818	0.0627	−129.217

*Bold numbers are the values with the best statistics. *RMSE* is the root mean square errors, *MAE* is mean absolute errors, *EF* is the Nash-Sutcliffe efficiency, *AIC* is Akaike Information criteria, and *d* is the Willmott’s index of agreement. These comparative statistics were obtained for the LR model as *RMSE* = 0.0926, *MAE* = 0.0719, *EF* = 0.532, *AIC* = −122.032, and *d* = 0.7479.

Based on the LR model, the logistic map function is computed as:

2 $$\begin{array}{rcl}f({\boldsymbol{\beta }}X) & = & -0.233+3.30\times {10}^{-4}BS+1.04\times {10}^{-7}LH+6.67\times {10}^{-7}BO\\  &  & -\,1.16\times {10}^{-4}AN+3.35\times {10}^{-6}P2+7.81\times {10}^{-6}AG+3.07\times {10}^{-11}ED\end{array}$$

where, BS: BSA-1 (µg/ml), LH (ng/ml), BO: BOEC (0 or 1), AN: ANGII (ng/ml), P2: PGE2 (ng/ml), AG: AGP (ng/ml), and ED: EDN-1 (ng/ml). Also, the comparative statistics are obtained for LR model as *RMSE* = 0.0926, *MAE* = 0.0719, *EF* = 0.532, *AIC* = −122.032, and *d* = 0.7479.

**Figure 1:**
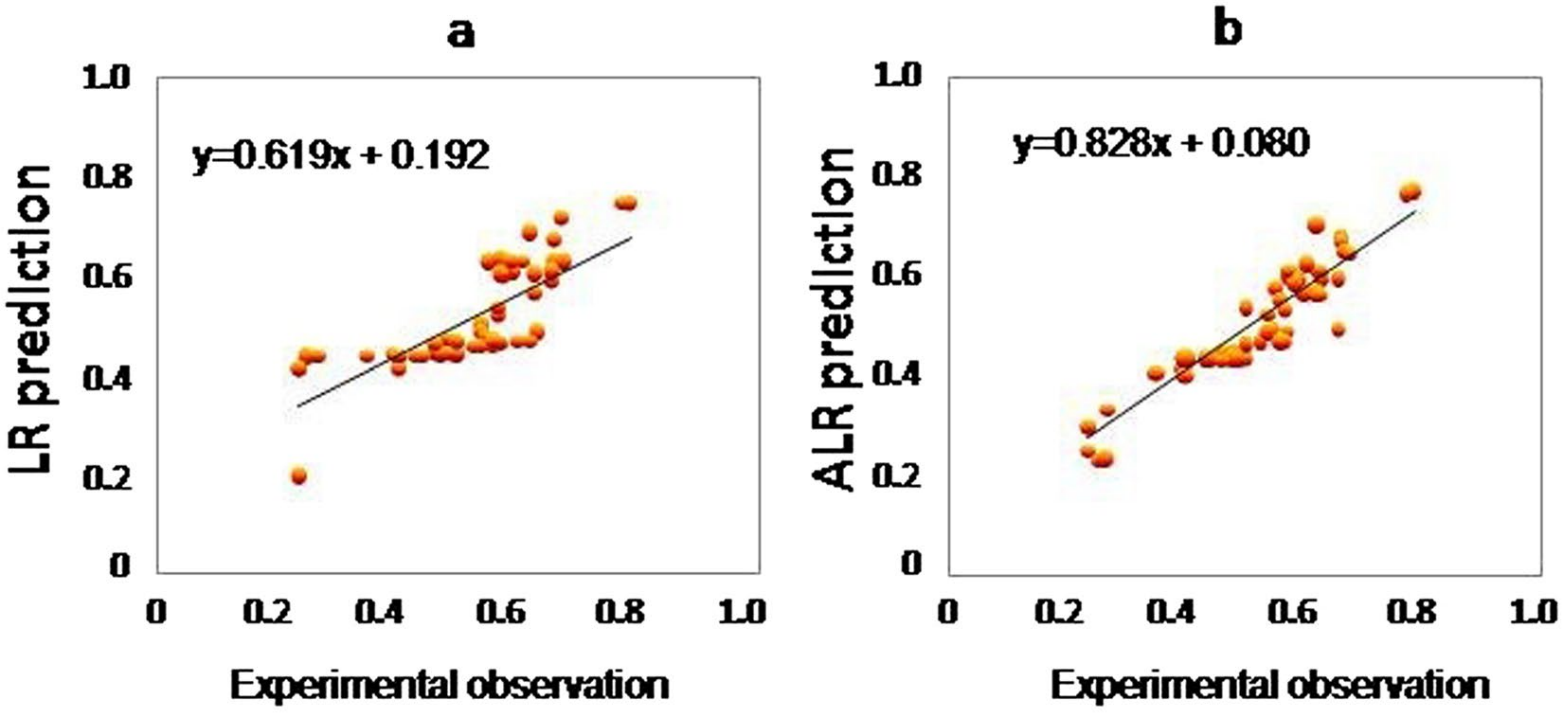
Scatterplots of the observed *versus* predicted data of spermophagy using the LR model (**a**) or the ALR model (**b**) for training data set. The training data consisted of 71.8% of the data, 58 data. Since the entering values were unavailable, we input end-experimental values for making scatter plots. Indeed, each end point data was the mean of individual experiment repeated for 3–8 times. SP, spermophagy.

The scatter plots of the predictions-based LR (in equation 2) or ALR (in equation 1) and experiments are shown in Fig. 1, (for 58 training data points from Table 2, since the entering values were unavailable, we input end-experimental values for making scatter plots). Regarding Fig. 1, and equation (1), the ALR model estimates fit the spermophagy data remarkably well. Scatter plots show the linear correlations for the LR model as R^2^ = 0.62 and the ALR model as R^2^ = 0.83, thus better predictions can be given the actual spermophagy data based on ALR model (around 33% improvement in correlation). This means that the ALR model has evaluated appropriate coefficients, based on the best penalty term (see Table 1), for the input variables (equation 1).

**Table 2:** Data and concentration of oviductal fluid components from the selected studies used for training the logit models.

Sources	Li (2010) 4 data	Marey (2014) 17 data	Marey (2016a) 12 data	Marey (2016b) 15 data	LIU (2014) 8 data
BSA(µg/ml)	4000				
LH (ng/ml)		0–10			
BOEC		0 or 1		0 or1	
ANGII (ng/ml)				0–10	
PGE2 (ng/ml)		0–352	0–35.2	0–35.2	0–3.52
AGP (ng/ml)					0–100
EDN-1 (pg/ml)			0–2490		
Phagocytosis	0.23–1.10	0.45–1.01	0.63–1	0.49–1.33	0.55–1

Equations 1 and 2 indicate that, respectively, BSA, AGP, PGE2, BOEC, LH, and EDN-1 show higher effects in the ALR model compared with the LR model. In addition, the negative effect of ANGII on reduction of spermophagy is highlighted better in the ALR model compare with the LR model. This might imply that the ALR model may overestimate these coefficients. But, regarding the lower relative error (Table 1) and the better prediction of scatterpoints (Fig. 1) obtained by the ALR model compared with the LR model, the estimate of these coefficients should be more accurate using the ALR model.

To validate our used models, the accuracy of the predicted spermophagy, using the logit models -based LR and ALR, was compared with the actual data (superoxide production data) (Table 3). The ALR model showed lower under-/over-prediction compared with the LR model (11.6 *vs* 18.9%; 10.2 *vs* 12.3%, respectively). These findings indicate that, on the basis of super oxide production data, the ALR model predicted spermophagy more efficiently than the LR model. The statistical errors for validation data based on the prediction of the logistic functions using the ALR and LR model are listed in Table 4. In comparison to the LR model, the ALR decreased the *RMSE* and *AIC* about 36% and 89%, repeatedly while improved the *EF* and Willmott’s index of agreement (*d*) about 30% and 19%, respectively. The relative mean error of the ALR model is obtained about 2 times less than the LR model. Thus, it can be concluded that the ALR improves the accuracy of the prediction of the logistic function and may be used to predict bovine oviductal spermophagy by PMNs *in vitro*.

**Table 3:** Validating the prediction of spermophagy using the LR and ALR models on basis of the experiments which evaluated Superoxide production as well.

Source	ANGII (ng/ml)	PGE2 (ng/ml)	AGP (ng/ml)	EDN-1 (pg/ml)	Phagocytosis	Superoxide	ALR	LR
Marey[2016a]	0	0	0	0	1	1	0.98723	0.90697
Marey[2016a]	0	0	0	2.49	0.92	0.96	0.91686	0.90659
Marey[2016a]	0	0	0	24.9	0.85	0.8	0.88349	0.90319
Marey[2016a]	0	0	0	249	0.75	0.73	0.82961	0.86905
Marey[2016a]	0	0	0	2490	0.63	0.64	0.51740	0.53188
Marey[2016a]	0	35.2	0	0	0.68	0.53	0.79128	0.84834
Marey[2016a]	0	0	0	249	0.82	0.53	0.93489	0.90319
Marey[2016a]	0	35.2	0	249	0.68	0.47	0.82746	0.84452
LIU[2014]	0	0	100	0	0.59	0.82	0.54977	0.52432
Marey[2016b]	0	0	0	0	1.33	1.43	1.37332	1.39558
Marey[2016b]	10	0	0	0	1.33	1.39	1.12946	0.96378
Marey[2016b]	1	0	0	0	1.3	1.4	0.99352	0.91269
Marey[2016b]	0.1	0	0	0	1.27	1.27	1.09679	0.90754
Marey[2016b]	0.01	0	0	0	1.26	1.32	1.09735	0.91269
Marey[2016b]	0.1	35.2	0	0	0.8	0.61	0.79770	0.85411

**Table 4:** Comparative statistics for the logistic functions-based LR and ALR in validation data.

Model	EF	d	RMSE	MAE	AIC	Tot-Phg*	Mean*	SD*	Rel-err*
ALR	0.774	0.920	0.132	0.103	−14.363	13.73	0.92	0.21	3.68
LR	0.542	0.775	0.207	0.163	−7.594	13.18	0.88	0.19	7.48

*The extracted data from the literature have the total phagocytosis (Tot-Phg), average (mean), standard deviation (SD) as 13.9, 0.95, and 0.27, respectively.

Based on Eq. (14), The *AICs* ^28^ for two models with and without END-1 are computed using autoregressive logistic regression as −130.457 and −129.792, respectively (Table 1). It can be obtained Δ*AIC* = 0.97 which is less than chi-square statistics with *k* = 1 and P = 0.05 i.e. 1.84, thus hypothesis *β*_8_ = 0 is accepted^29^. Thus, using Akaike indicator, END-1 has been shown a non-significant affect on spermophagy compared with the other input variables. Thus, this input variable can be removed from the logit model in the next investigations.

### Calculation of marginal effects to reveal effects of BOFCs on *in vitro* spermophagy by PMNs in cattle

**Figure 2:**
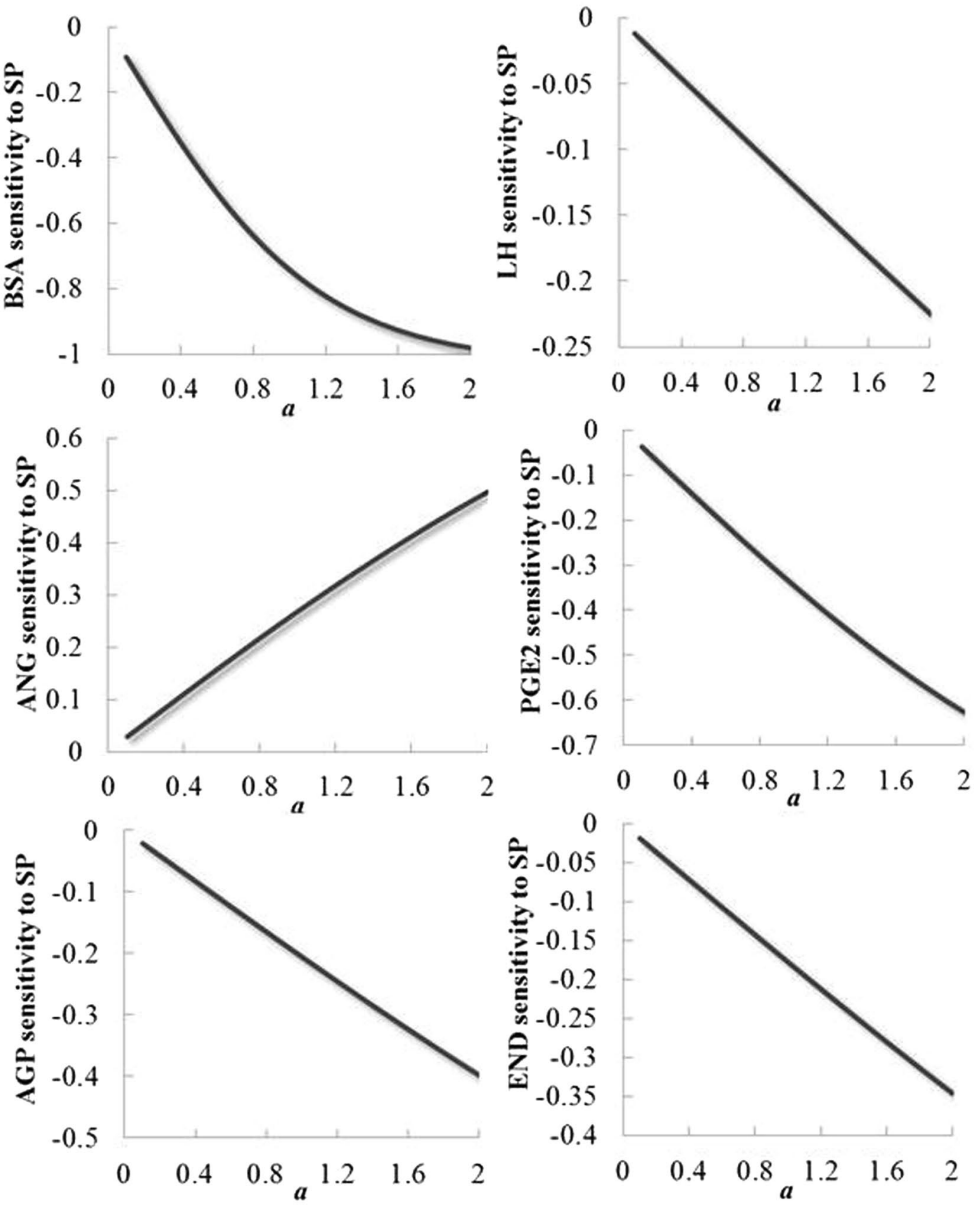
Marginal effects of the different input variables on the reduction in spermophagy.

To see how change in spermophagy is related to changes in the input variables, marginal effects are computed (Fig. 2). Indeed, in the dose-dependent experiments, which were selected in the present study, the concentration of variables had been increased at the 10-fold intervals. So, the marginal effects were computed to reveal the instantaneous rate of change in smaller intervals (one tenth-fold intervals, 2 is considered as the maximum concentration of each variable that was used in the actual experiments). The results confirmed the dose-dependent reduction of spermophagy by BOFCs (except BSA) based on the predictions of the ALR model. Also, the marginal effects showed that BOFCs seem to be effective in reducing spermophagy at levels above concentrations used in actual *in vitro* experiments. However, when we computed slopes and marginal effects of BOFCs, as shown in Fig. 2, the marginal effect for BSA showed that BSA reduces spermophagy stronger. But, its slope line graph is not as similar as other factors and shows slightly a curved shape rather than a straight shape. This means that all BOFCs except BSA show a dose-dependently effect on spermophagy (0.1 to 0.5) while BSA from a given point (1.5 to 2.5) fails to reduce spermophagy. These findings confirmed the data from actual experiment in which PGE2, EDN-1, and AGP had dose-dependently reduced and ANGII had dose-dependently increased spermophagy by PMNs^8, 19–21^.

### Prediction of the reciprocal effects of BOFCs on *in vitro* spermophagy by PMNs in cattle

**Figure 3:**
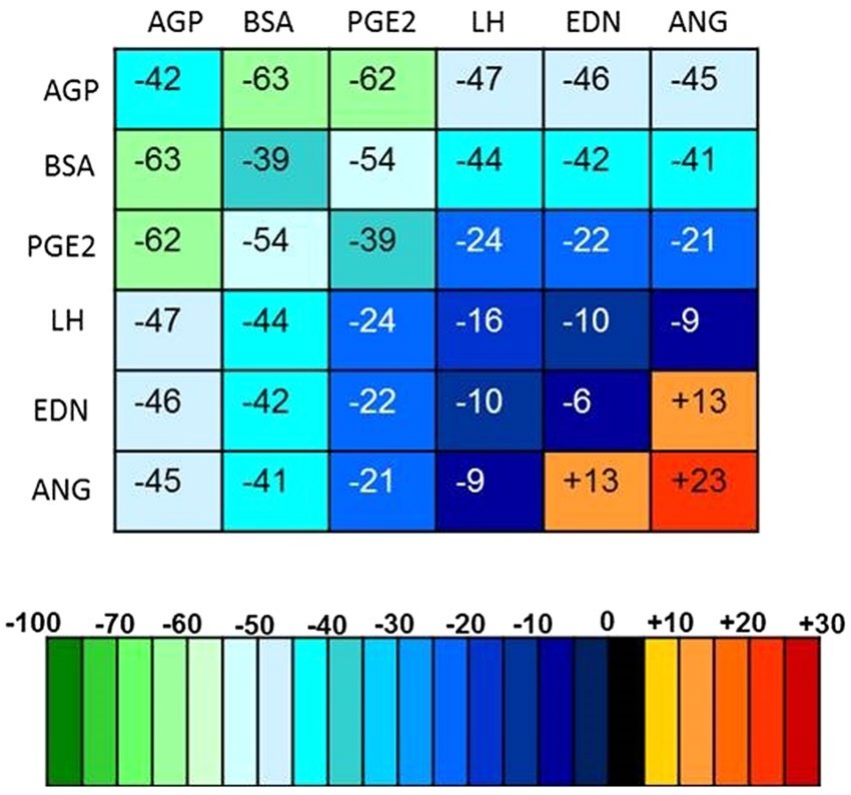
The prediction of changes in spermophagy by each oviductal fluid component and by reciprocal interactions using the ALR model (negative values mean reduction and positive values mean increase in spermophagy in comparison with the control group.

As mentioned above, among BOFCs, ANGII enhances spermophagy. Therefore, we tried to use the ALR model to predict the reciprocal effects of the BOFCs on the reduction of spermophagy and to reveal that to what extend the BOFCs are able to abrogate ANGII-induced spermophagy. Figure 3 shows both individual and reciprocal effects of BOFCs on percentage of reduction (negative values) or increase (positive values) of sperm phagocytosis compared to the control group. The combination of AGP or BSA with other BOFCs produced an average reduction of 52.5 and 48.5% in spermophagy, respectively, named here as class-1 factors. As class 2, the model predicted that the combination of PGE2 with other factors shows an average reduction of 36.6% in spermophagy. As class 3, the combination of LH or EDN-1 with other factors reproduces an average reduction of 26.8 and 26.6% in spermophagy. Finally, the ALR model used to determine the strongest BOFCs that abrogate the increasing effect of ANGII on spermophagy by PMNs. The ALR model demonstrates that BSA, AGP, PGE2, or LH, respectively, abrogates ANGII-increased spermophagy by PMNs.

## Discussion

Many factors may affect fertility in mammals. Indeed, the likelihood of full-term pregnancies in inseminated cows is only 50%^30^, implying potential large economic losses. Spermatozoa are able to activate neutrophils^12^, which are able to trigger the phagocytosis of these male gametes and hinder their motility^9, 12^. Alternatively, many of mammalian sperm are attacked and phagocytosed by leukocytes and epithelial cells in the FRT^14–16^. Isthmic epithelial cells and leukocytes are believed to involve in phagocytosis of surplus/post-capacitated sperm that entered the oviduct and did not contribute in fertilization^15^, implying a physiological role for spermophagy process in regulating possible sever inflammation that may be resulted from dead/remained post-capacitated sperm^15, 16^. These suggest that spermophagy can be considered as a positive and even essential event for a normal fertilisation and early embryonic development in oviduct^16^. On the other hand, it has been shown that freshly capacitated sperm cells dose-dependently (being significant at levels more than 10^5^ sperm/ml), but not dead or abnormal ones, induce PGE2 secretion by bovine oviductal epithelial cells^17^. Also, PGE2 has been shown to dose-dependently reduce phagocytosis of freshly capacitated sperm by PMNs in bovines^8^, indicating a cross-talk between number of capacitated sperm cells and oviductal epithelial cells to regulate spermophagy. Alghamdi *et al*.^31^ reported that incubation of equine sperm with either seminal plasma or isolated proteins from seminal plasma reduced sperm binding to PMNs and improved fertility. Very recently, Zambrano *et al*.^32^ showed that human sperm cells act as a sufficient stimulus to induce the neutrophil extracellular traps (NETs) formation that may result in reduced motility and fertility. However, further studies are still necessary to determine signals produced by post-capacitated/remained post-fertilization or freshly capacitated sperm toward PMNs. The possible responses to these questions might help endangered or threatened species, economic animals and humans^32, 33^.

Recently, other oviductal fluid components such as EDN-1, ANGII, AGP, and BSA, have been shown to regulate *in vitro* spermophagy by PMNs in cattle. Importantly, these factors may raise interactions when they release together and influence their own individual responses. Unfortunately, there are still a few studies addressing this issue, more investigations are needed to determine the proportion of each factor or their combinations in regulating bovine spermophagy. Indeed, revealing such issues may help to design next experiments more efficiently, at first. Moreover, knowing the contribution level of each factor in reducing or enhancing spermophagy as well as their combination may, at least in part, help a better understanding of physiological events and the prediction of spermophagy by PMNs, based on *in vitro* experiments.

Recently, computer-based systems have been used to clinical use for medical diagnosis, decisions, outcome prediction, and even patient care^34–36^. Most experiments in this field are done dose-dependently to reveal responses and such dose-response models include a range of statistical models such as nonlinear regression. We, therefore, investigated the ALR and LR models to determine which of them fits better to the actual dose-dependent data and provide more accurate predictions of individual responses and reciprocal interactions. The autoregressive logistic regression used in the present study allows the analysis and interactions to be performed and modeled easily and quickly on any standard desktop computer in addition to this fact that its statistical theory is very well characterized^37^. However, as an important disadvantage, this model needs a very large sample size to accurately estimate the parameters when there are many independent variables^37^. But, our results suggest that the ALR may improve correlation and resultant predictions without using a lot of data; this phenomenon can be explained by the fact that, in this study, the interaction effects are relatively greater than the main effects^38^.

By comparison, the ALR model showed the highest correlation and lowest errors *RMSE* and *AIC* compared to LR for prediction of reducing spermophagy. This means that the ALR model outperforms the LR model in prediction accuracy. Only a few *in vitro* data were used to predict the spermophagy level under the effect of the oviductal fluid components. However, the results indicated that the ALR model is a suitable model to predict and classify these influencing spermophagy components applied dose dependently in the actual *in vitro* experiments. With regard to the ALR model, we identified and ranked the oviductal fluid factors associated with spermophagy such that AGP, PGE2, LH, and EDN-1 reduce spermophagy by neutrophils (42, 39, 16, and 6%, respectively) and ANGII increases (23%) as observed in the actual experiments. The calculation of Akaike indicator, *AIC*, showed that the role of EDN-1 is not significant in the ALR model and subsequently in spermophagy and can be removed from the model. But, EDN-1 is still illustrated in Fig. 3, since we would like to show and classify the role of all bovine oviductal fluid components on spermophagy as much as possible.

Recently, Liu *et al*.^19^ showed that AGP concentration was numerically decreased in bovine oviductal flushes during the estrous cycle with the highest level at the pre-ovulatory stage. As mentioned, the ALR model predicted that AGP, individually or in combination with other factors, has the highest impact on spermophagy. They also reported that AGP dose-dependently reduced spermophagy. It seems that the highest AGP concentration at the pre-ovulatory stage might result in a reduced phagocytosis of upcoming sperms. Possibly, reduction of AGP concentration after ovulation may lead to enhance spermophagy at this stage. This, in turn, may support the elimination of surplus/remained post-fertilization sperm in bovine oviduct. The other factors like PGE2 dose-dependently reduced spermophagy^8^. Importantly, PGE2 showed its highest level under LH stimulation, LH surge during the pre-ovulatory stage^8, 39^. This might imply that high concentrations of AGP, EDN-1 and PGE2 observed during pre-ovulatory stage may involve in reducing phagocytosis of upcoming sperm. On the other hand, when their concentrations are reduced at the post-ovulatory stage, the reducing effect of these factors on spermophagy would be alleviated. This may support the hypothesis of the elimination of remained sperm in providing a timely controlled environment for the early embryo development in cattle.

To examine how changes in the input variables i.e. different doses of BOFCs affect changes in spermophagy, marginal effects were computed which reveal the instantaneous rate of changes in smaller intervals (one tenth-fold intervals compared with10-fold intervals that had been used in *in vitro* experiments). Based on the ALR model, marginal effects were computed and showed that all BOFCs except BSA showed a dose-dependent effect on spermophagy as their slope lines are straight. In case of BSA, the marginal showed that BSA reduces spermophagy stronger, but its slope line graph is not as similar as other factors and trends a curved shape rather than a straight shape. This means that BSA from a given dose fails to reduce spermophagy and show not a dose-dependent effect. These marginal effects confirmed the *in vitro* data of actual experiments in which PGE2, EDN-1, and AGP had dose-dependently reduced and ANGII had dose-dependently increased spermophagy by PMNs^8, 19–21^.

The ALR model additionally enables us to classify these factors as well. The ALR model also predicted the reciprocal effect of oviductal fluid components on *in vitro* spermophagy by PMNs in bovines. It might be proposed that a computer-based predictive model, in this case the ALR model, might be used to facilitate the early identification of cases at a high risk of spermophagy after insemination or intercourse, and to design next experiments more efficiently. Of course, this hypothesis shows a strong limitation that the correlation between serum and oviductal concentrations of selected factors in this study has not been yet addressed. This issue makes the prediction of spermophagy on the basis of serum markers much harder. Also, because of complexity of *in vivo* models with a lot of interactions, the establishment of *in vivo* relevant *in vitro* system is difficult. However, *in vitro* models have substantially encouraged the understanding of biological mechanisms. Therefore, further *in vivo*/*in vitro* studies are required to specify the interactions between spermophagy/survival and oviductal fluid components in bovine or other species and also to evaluate the correlation between *in vitro* and *in vivo* models.

## Methodology

### Data set

The data were obtained from 5 *in vitro* studies that were conducted in Japan^2, 8, 19–21^. These studies had evaluated the effect of BOFCs on *in vitro* spermophagy by PMNs.

Two studies used a single dose of bovine serum albumin, BSA, (0.4%, w/v) or luteinizing hormone (LH, 10 ng/ml) and 4 dose-dependent studies used PGE2 (0–352 ng/ml; 97.68 ± 170.28 ng/ml; mean ± standard deviation(SD)), AGP (0–100 ng/ml; 32.2 ± 43.06 ng/ml; mean ± SD), ANGII (0–10 ng/ml; 2.22 ± 4.37 ng/ml; mean ± SD), or EDN-1(0–24.9 ng/ml; 5.53 ± 10.87 mean ± SD). A summary of the ranges of different BOFCs used in above studies is given in Table 2. The concentrations of PGE2, EDN-1, ANGII, and AGP had been measured directly in the bovine oviduct flush and in the BOEC-conditioned media using double-antibody enzyme immunoassays (EIA), as previously described. Generally, EIA was done in the 96 wells ELISA plate (Corning, New York, NY, USA) which coated with 50 µg second antibody (anti rabbit IgG, Seikagaku Co. Tokyo, Japan). For EIA determination, first, 30 µl of standards or samples were incubated with 100 µl of their corresponding polyclonal antibody (1:250,000) at 4 °C for 24 h. Then, samples were decanted and incubated with 100 µl of PGE2-HRP (1:30,000)^40^ for further 2 h at 4 °C. The values of coefficients of variance within and between assays for PGE2 were 7.3 and 11.4% respectively.

To determine EDN-1and ANGII concentrations in both oviductal flush (17 oviducts from different phases of the estrous cycle, pre-ovulatory, post-ovulatory, and mid luteal phases) and BOEC culture supernatants (12 experiments), freshly isolated oviducts were gently flushed with PBS−/− (0.2 ml/oviduct), and the resultant fluid was collected in a sterile tube. For EDN-1 determination^20^, a commercial EIA kit (Cayman, Ann Arbor, MI, USA) was conducted according to the manufacture’s protocol. The intra- and inter-assay coefficients of variation for EDN-1 were 7.8% and 10.7%, respectively.

ANG II concentrations were measured using an antibody enzyme immunoassay as reported before^41^. The intra- and inter- assay coefficients of variation for ANG II were 3.9% and 8.4%, respectively.

AGP concentrations were determined using an ELISA kit (Uscn Life Science, Wuhan, China) according to the manufacturer’s manual. The optical density (OD) was measured at 450 nm using an ELISA reader (Multiskan MS plate reader, Thermo Labsystems, Vantaa, Finland). The values of coefficients of variance within assay and between assays for AGP were 10 and 12% respectively.

The ED50 values of the assays were 260 pg/ml for PGE2, 115 pg/ml for EDN-1, and 150 pg/ml for ANGII. The standard curves ranged from 20–20000 pg/ml for PGE2, 3.9 to 250 pmol/ml for EDN-1, 2.4 to 5000 pg/ml for ANGII, and 15.5 to1000 ng/ml for AGP. The concentration of LH was based on its pre-ovulatory peak in circulation^40, 42^.

Four out 5 selected studies^8, 19–21^ were carried out at the Field Center of Animal Science and Agriculture of Obihiro University, and blood collection and experiments were done in compliance with the Guidelines for the Care and Use of Agricultural Animals at Obihiro University. Across all mentioned studies, PMNs were isolated from the blood of multiparous Holstein cows during the luteal stage. Collected blood was mixed with PBS−/− and layered with Ficoll-Paque solution (Lymphoprep, Axis Shield, Oslo, Norway), and centrifuged at 1000 g for 30 min at 10 °C. after mixing PMN layer with ammonium chloride lysis buffer (NH4Cl, 155 mM; KHCO3, 3.4 mM; and EDTA, 96.7 mM) for 10 s, the mixture was centrifuged at 500 g for 10 min at 10 °C to isolate PMNs from red blood cells. The 4-h pre-incubated neutrophils (with each of BOFCs) mixed with TL-HEPES and incubated with freshly capacitated sperm and serum in a 96-well untreated polystyrene microtest plate (Thermo Scientific, Roskilde, Denmark) for 60 min with gentle swirling on a test-plate shaker. The final concentrations of PMNs, sperm, and fresh serum were 8 × 10^6^, 4 × 10^6^ cells/ml, and 12% (v/v) respectively. After incubation, to dissociate PMNs agglutination, heparin (40 mg/ml in TL-HEPES) was used to the suspension of PMNs and sperm. Then, by adding 25 µl of 2% (v/v) glutaraldehyde to 75 µl of samples, samples were fixed and then mounted on the glass slides and examined for the percentage of PMNs with phagocytosed sperm (at least, 400 PMNs were counted across different parts of the specimen) that was recorded as phagocytosis rate. In parallel with PMNs preparation, the sperm capacitation was carried out using modified Tyrod’s albumin, lactate, and pyruvate medium (Sp-TALP)^43^ in a 96-well untreated polystyrene microplate. Briefly, 1-h swim-up sperm was suspended in Sp-TALP supplemented with 10 mg/ml heparin (50 × 10^6^ sperm/ml) and incubated for 4 h. To confirm capacitation, lysophosphatidylcholine (100 mg/ml for 15 min) was used to induce acrosome reaction which, in turn, was determined by a dual staining procedure with Trypan Blue supravital stain and Giemsa^44^. Frozen semen straws from a single ejaculate were obtained from the highly fertile Holstein bulls of Genetics Hokkaido Association (Hokkaido, Japan). Importantly, it should be mentioned that heparin that is commonly used to induce sperm capacitation has been shown to reduce phagocytotic and chemotactic activities of PMNs against sperm^2^. Li and Funahashi^2^ reported that 100 µg/ml heparin is enough to reduce spermophagy. In the present study, other *in vitro* studies, except Li and Funahashi study, that had been selected, did not exclude this effect of heparin in their experiments^8, 19–21^. However, in these studies^8, 19–21^, only 10 µg/ml of heparin was used for sperm capacitation.

Finally, the authors of mentioned studies repeated *in vitro* experiments three to eight times, analyzed the data using Stat-View 5.0 (SAS Institute, Inc., Cary, NC, USA), one-way ANOVA followed by multiple comparison tests (Fisher’s test for three groups, and Bonferroni’s test for more than three groups), and reported results as mean ± SEM. Since the original data were not available, we had to use mean values of each experiment, end-experimental data, instead of entering values.

### Logistic regression (LR) model

The logit modeling is commonly implemented to calibrate an event in which the logistic function i.e. *pi* is used to predict by the following its expected outcome formula^45, 46^:

3 $$E(yi|{\boldsymbol{X}}i,{\boldsymbol{\beta }})=pi=\frac{\exp [f({\boldsymbol{\beta }}Xi)]}{1+\exp [f({\boldsymbol{\beta }}Xi)]}$$

where, ***β*** is the vector of the unknown coefficients, ***X*** is the input variables vector, *yi* is the observed spermophagy and *exp* is the exponential function. The mean effort in the logistic regression (LR) is to determine the unknown coefficients based on the effect of BOFCs on spermophagy by PMNs. The logistic transformation is used to simple calibration in logit model as follows:

4 $$f({\boldsymbol{\beta }}X)=\,\mathrm{ln}(\frac{p}{1-p})={\beta }_{0}+{\beta }_{1}{x}_{1}+{\beta }_{2}{x}_{2}+{\beta }_{3}{x}_{3}+\mathrm{...}+{\beta }_{8}{x}_{8}$$

where, *ln* is logarithmic function, *p* the probability distribution of logistic function, and *β*_0_ − *β*_8_ are the regressed coefficients of logit model, where these coefficients are determined based on the training data form Table 2 using the MATLAB software (Version 7.10.0; The MathWorks Inc., Natick, Massachusetts, USA, 2010)^47^.

### Autoregressive logistic regression (ALR) model

The accurate predictions of the mathematical models-based LR or ALR are more important issues to analyze and classify factors regulating spermophagy. The LR is simpler than the ALR model but it may provide inaccurate predictions. The LR model provides error terms with the mean of zero and each input variable is also considered with a same influence in the modeling approach-based logistic function in equation (3). Consequently, the LR model may produce an inappropriate estimation of spermophagy. Since, the effects of each input variables such as ANGII, EDN-1, PGE2, LH, and BSA are considered with a same weight. This means that each data and variables have a similar importance in regression process. Therefore, the heterogeneity in the input variables is effectively increased in the logit model-based LR. On the other hand, the ALR model is calibrated based on a nonlinear regression procedure that may produce an accurate prediction in order to use an autoregressive term in the logit model to consider the effective input variables for predicting the regulating spermophagy. Thus, an iterative formula is proposed for calibration of the ALR model based on a dynamic search direction to achieve the stabilization of the calibrated coefficients of the model. So, the ALR model seems to be an effective modeling to predict spermophagy based on the different capacity of BOFCs (input variable) in regulating spermophagy by PMNs.

The maximum likelihood estimator is defined to approximate the unknown coefficients using the ALR as follows^46^:

5 $$M({\boldsymbol{\beta }})=\sum _{i=1}^{n}\mathrm{ln}[pi]\,yi+\,\mathrm{ln}[1-pi](1-yi)-\lambda {\Vert {\boldsymbol{\beta }}\Vert }^{2}$$

In which, *n* is the number of data point, *pi* is the logistic function at *i*_*th*_ input data points (***X*** *i*) and coefficient vector ***β***. $\lambda {\Vert {\boldsymbol{\beta }}\Vert }^{2}$ is a penalty term, which is added to obtain a better generalization. A flexible model using ALR in equation (5) can be provided based on the influence of each input variable by the penalty term in terms of each unknown coefficient (in the LR model, *λ* is zero). Indeed, penalty term should be minimized that this, in turn, maximizes the likelihood of prediction, based on the minimum length of the vector in the equation (5) that should be maximized.

Considering that BOFCs have various effects on the phagocytic activity of PMNs, some reduce phagocytosis to a greater extent and some reduce that to a lesser extent, and the modeling approach using the LR model is very simple giving same size to all input variables, a model is needed to overcome this problem. As shown in equation (5), the calibration data using the ALR model are to develop and iterative procedure to estimate the unknown coefficients of logit model in equation (4) i.e. *f*(***βX***). Indeed, ALR model considers various input variables with different degrees of effect. Thus, to minimize the penalty term, the ALR model gives a greater or smaller coefficient to variables regarding their higher or lower impacts on spermophagy, respectively.

The iterative method using steepest descent search direction can be used to determine the unknown coefficients of the ALR model as follows:

6 $${{\boldsymbol{\beta }}}_{k+1}={{\boldsymbol{\beta }}}_{k}+{\alpha }_{k}\nabla M({{\boldsymbol{\beta }}}_{k})$$

where, ***β***_*k*+1_ and ***β***_*k*_ are the new and pervious coefficient vector, *α*_*k*_ is the step size at each iteration. ∇*M*(***β***_*k*_) is the gradient vector of the maximum likelihood function at the coefficient vector ***β***_*k*_, which is determined based on the equation (5) as follows:

7 $$\nabla M({{\boldsymbol{\beta }}}_{k})=\sum _{i=1}^{n}\frac{\partial \,\mathrm{ln}\,[pi]}{\partial {\boldsymbol{\beta }}}{|}_{{\boldsymbol{\beta }}={{\boldsymbol{\beta }}}_{k}}yi+\frac{\partial \,\mathrm{ln}\,[1-pi]}{\partial {\boldsymbol{\beta }}}{|}_{{\boldsymbol{\beta }}={{\boldsymbol{\beta }}}_{k}}(1-yi)-2\lambda {{\boldsymbol{\beta }}}_{k}$$

Based on the coefficient ***β***_*k*_, we can be obtained a real value for the gradient vector in terms of each coefficient. The above iterative formula depends on the step size, which is dynamically adjusted as follows:

8 $${\alpha }_{k+1}={\delta }_{k}{\alpha }_{k}$$

In which, *α*_0_ = *α*_1_ = 1 and *δ*_*k*_ is the adaptive coefficient, which dynamically adjusts the step size at each iteration, as follows:

9 $${\delta }_{k}=\,{\rm{\max }}(0.95,\frac{\Vert {{\boldsymbol{\beta }}}_{k}-{{\boldsymbol{\beta }}}_{k-1}\Vert }{\Vert {{\boldsymbol{\beta }}}_{k}-{{\boldsymbol{\beta }}}_{k+1}\Vert })$$

It can be concluded from the equation (8) that *α*_*k*+1_ ≤ *α*_*k*_ thus in *k* → ∞, *α*_*k*_ ≈ 0. This means that a stable result is obtained using the proposed iterative formula to determine unknown coefficients, thus it captured a fixed coefficient for this logit model-based ALR in equation (5) i.e. ***β***_*k*+1_ ≈ ***β***_*k*_ when *k* → ∞ because *α*_*k*_ ≈ 0. The proposed search direction with the dynamic step size in equation (8) is simply formulated and we can be coded based on the below steps in a computer program.

**Step1:** Set *k* = 0, *α*_0_ = *α*_1_ = 1, stopping criterion of *ε* = 10^−5^, and initial coefficient of ***β***_0_ = 1

**Step 2:** Compute the gradient vector of the maximum likelihood estimator at coefficient ***β***_*k*_ i.e. ∇*M*(***β***_*k*_) based on equation (7)

**Step 3:** Determine the new coefficients in terms of equation (6)

**Step 4:** For *k* > 1, determine the adaptive coefficient using equation (9)

**Step 5:** For *k* > 1, compute the new step size based on the equation (8)

**Step 6:** Check the convergence as $\Vert {{\boldsymbol{\beta }}}_{k+1}-{{\boldsymbol{\beta }}}_{k}\Vert  < \varepsilon$, if satisfies, then given ***β***_*k*+1_ and stop, else, Set *k* = *k* + 1 and go to **Step 2**.

The iterative formula to determine the unknown coefficients is used for calibrating the logistic function using ALR to predict spermophagy using input variables.

### Comparative criteria

The spermophagy-related data are transformed as $R{P}_{i}=0.2+\frac{1.4-SPi}{2}$ in which *RP*_*i*_ is mapping spermophagy and *SPi* is reducing spermophagy for *i*_*th*_ data. This mapping can show the positive effects of BOFCs on the reduction of phagocytosis using a new data interval 0 to 1. The logit models-based NLR for different penalty factor *λ* are regressed using the proposed iterative formula to calibrate the unknown coefficients. Then, their predictions of spermophagy are compared by the following comparative error statistics^48^:

10 $$RMSE=\sqrt{\frac{\sum _{i=1}^{n}(yi-pi{)}^{2}}{n}}$$

11 $$MAE=\frac{\sum _{i=1}^{n}|yi-pi|}{n}$$

12 $$NSE=1-\frac{\sum _{i=1}^{n}(yi-pi{)}^{2}}{\sum _{i=1}^{NE}(yi-\bar{y}{)}^{2}}\,\,-\infty \le EF\le 1$$

13 $$d=1-\frac{\sum _{i=1}^{n}(yi-pi{)}^{2}}{{\sum _{i=1}^{n}(|yi-\bar{y}|+|pi-\bar{p}|)}^{2}}\,\,0\le d\le 1$$

14 $$AIC=-2Ln[M({{\boldsymbol{\beta }}}^{{\boldsymbol{\ast }}})]+2C$$

In which, *RMSE* is the root mean square errors, *MAE* is mean absolute errors, *NSE* is the Nash-Sutcliffe efficiency, *d* is the Willmott’s index of agreement, and *AIC* is Akaike Information criteria (*AIC*)^28^. *RMSE* and *MAE* show the average difference between predicted (_*pi*_) and observed spermophagy (_*yi*_).The lower values for *RMSE*, *MAE* and *ACI* indicate a better fitness. $\bar{y}$ and $\bar{p}$ are the mean (average) of the observed and predicted data, respectively. Both *NSE* and *d* show the correlation between the predicted and observed data. The larger value for the agreement index indicates a better agreement for prediction so that _*d*=0_ indicates null agreement (no correlation) and _*d*=1_ indicates total agreement (perfect fit).

AIC is an alternative criterion to select proper input variables, considering the importance of variable instead of influence effects on the models. The *AIC* was computed for logit model based on the maximum likelihood value at the coefficient vector ***β*** * = {*β*_1_, *β*_2_, …, *β*_*i*_,…, *β*_*m*_} in which, *m* is number of the coefficients (eight coefficients in this study). The null hypothesis of a variable (*x*_*i*_) as *β*_*i*_ = 0 with the maximum likelihood value at the coefficient vector ${\hat{{\boldsymbol{\beta }}}}^{\ast }=\{{\beta }_{1},{\beta }_{2},\mathrm{...},{\beta }_{m}\}$ can be tested using *P*-values and *AIC* by Murtaugh’s relation as follows^29^:

15 $$P=\Pr ({\chi }_{k}^{2} > {\rm{\Delta }}AIC+2k)\Rightarrow {\rm{\Delta }}AIC={F}_{{\chi }_{k}^{2}}^{-1}(1-P)-2k$$

where, *P* is the *P*-value for null hypothesis *β*_*i*_ = 0 which indicates to remove variable *x*_*i*_ (e.g. END-1) from the logit model and *k* is the number of coefficient for the variable *x*_*i*_ in logit model that it is 1 and ${\chi }_{k}^{2}$ is chi-square distribution function with *k* degrees of freedom which is obtained 1.84 for *k* = 1 and P = 0.05^29^. Δ*AIC* is explained as the changes in Akaike’s information criterion based on the all input variables and null variable *x*_*i*_. If Δ*AIC* is less than the chi-square statistics (e.g. 1.84 for *k* = 1 and P = 0.05) then the variable *x*_*i*_ can be removed from the modeling input data set.

## References

[1] Kölle S (2015). Transport, distribution and elimination of mammalian sperm following natural mating and insemination. Reprod. Dom. Anim..

[2] Li JC, Funahashi H (2010). Effect of blood serum, caffeine and heparin on *in vitro* phagocytosis of frozen-thawed bull sperm by neutrophils derived from the peripheral blood of cows. Theriogenology.

[3] Lopez-Gatius F (2000). Site of semen deposition in cattle: a review. Theriogenology.

[4] Suarez SS (2007). Interactions of spermatozoa with the female reproductive tract: inspiration for assisted reproduction. Reprod. Fertil. Dev..

[5] Vishwanath R (2003). Artificial insemination: the state of the art. Theriogenology.

[6] Mitchell JR, Senger PL, Rosenberger JL (1985). Distribution and retention of spermatozoa with acrosomal and nuclear abnormalities in the cow genital tract. J. Anim. Sci..

[7] Sostaric E (2008). Sperm binding properties and secretory activity of the bovine oviduct immediately before and after ovulation. Mol. Reprod. Dev..

[8] Marey MA (2014). Bovine oviduct epithelial cells downregulate phagocytosis of sperm by neutrophils: prostaglandin E2 as a major physiological regulator. Reproduction.

[9] Alghamdi AS (2009). Species-specific interaction of seminal plasma on sperm–neutrophil binding. Anim. Reprod. Sci..

[10] Mattner PE (1968). The distribution of spermatozoa and leucocytes in the female genital tract in goats and cattle. J. Reprod. Fertil..

[11] Strzemienski ,PJ (1989). Effect of bovine seminal plasma on neutrophil phagocytosis of bull spermatozoa. J. Reprod. Fertil..

[12] Alghamdi AS, Foster DN (2005). Seminal DNase frees spermatozoa entangled in neutrophil extracellular traps. Biol. Reprod..

[13] Haney AF, Misukonis MA, Weinberg JB (1983). Macrophages and infertility: oviductal macrophages as potential mediators of infertility. Fertil. Steril..

[14] Murakami M, Nishida T, Shiromoto M, Iwanaga S (1985). Phagocytosis of spermatozoa and latex beads by epithelial cell of the cat oviduct: combined SEM and TEM study. Arch. Histol. Jpn..

[15] Chakraborty J, Nelson L (1975). Fate of surplus sperm in the fallopian tube of the white mouse. Biol. Reprod..

[16] Eisenbach M (2003). Why are sperm cells phagocytosed by leukocytes in the female genital tract?. Med. Hypotheses.

[17] Kodithuwakku SP, Miyamoto A, Wijayagunawardane MPB (2007). Spermatozoa stimulate prostaglandin synthesis and secretion in bovine oviductal epithelial cells. Reproduction.

[18] Yousef MS (2016). Sperm binding to oviduct epithelial cells enhances TGFB1 and IL10 expressions in epithelial cells as well as neutrophils i*n vitro*: prostaglandin E2 as a main regulator of anti- inflammatory response in the bovine oviduct. PloS one.

[19] Liu J (2014). An acute-phase protein as a regulator of sperm survival in the bovine oviduct: alpha 1-acid-glycoprotein impairs neutrophil phagocytosis of sperm i*n vitro*. J. Reprod. Dev..

[20] Marey MA (2016). Endothelin-1 downregulates spermophagy by neutrophils *in vitro*: a physiological implication in bovine oviduct immunity. J. Reprod. Dev..

[21] Marey MA (2016). Angiotensin II increases spermophagy by neutrophils *in vitro*: a possible physiological role in the bovine oviduct. Mol. Reprod. Dev..

[22] Haghighi M (2016). A comparison of rule-based analysis with regression methods in understanding the risk factors for study withdrawal in a pediatric study. Sci. Rep..

[23] Loley. C (2016). No association of coronary artery disease with X-chromosomal variants in comprehensive international meta-analysis. Sci. Rep..

[24] Sun H, Wang S (2012). Penalized logistic regression for high-dimensional DNA methylation data with case-control studies. Bioinformatics.

[25] Wu HY (2016). Predicting postoperative vomiting among orthopedic patients receiving patient-controlled epidural analgesia using SVM and LR. Sci. Rep..

[26] Zhang Q (2015). Selection of models for the analysis of risk-factor trees: leveraging biological knowledge to mine large sets of risk factors with application to microbiome data. Bioinformatics.

[27] Ritz C, Baty F, Streibig JC, Gerhard D (2015). Dose-response analysis using R. PloS one.

[28] Akaike H (1983). Information measures and model selection. International Statistical Institute.

[29] Lavine M (2014). Comment on murtaugh. Ecology.

[30] Peddinti D (2008). Comprehensive proteomic analysis of bovine spermatozoa of varying fertility rates and identification of biomarkers associated with fertility. BMC Sys. Biol..

[31] Alghamdi AS, Foster DN, Troedsson MHT (2004). Equine seminal plasma reduces sperm binding to polymorphonuclear neutrophils (PMNs) and improves the fertility of fresh semen inseminated into inflamed uteri. Reproduction.

[32] Zambrano, F. *et al*. Leukocytes co-incubated with human sperm trigger classic neutrophil extracellular traps formation, reducing sperm motility. *Fertil*. *Steril*. doi:10.1016/j.fertnstert.2016.06.005 (2016). 10.1016/j.fertnstert.2016.06.005 27344301

[33] Henry F (2015). Seminal fluid promotes *in vitro* sperm–oviduct binding in the domestic cat (*Felis catus*). Theriogenology.

[34] Hu YJ (2012). Decision tree-based learning to predict patient controlled analgesia consumption and readjustment. BMC Med. Inform. Decis. Mak..

[35] Johnston ME, Langton KB, Haynes RB, Mathieu A (1994). Effects of computer-based clinical decision support systems on clinician performance and patient outcome. A critical appraisal of research. Ann. Intern. Med..

[36] Lee SY, Hung CJ, Chen CC, Wu CC (2014). Survival analysis of postoperative nausea and vomiting in patients receiving patient controlled epidural analgesia. J. Chin. Med. Assoc.

[37] Gilbert-Diamond, D. & Moore, J. H. Analysis of gene-gene interactions. *Curr*. *Protoc*. *Hum*. *Genet*. **14**, doi:10.1002/0471142905.hg0114s70 (2011). 10.1002/0471142905.hg0114s70 PMC4086055 21735376

[38] Marchini J, Donnelly P, Cardon LR (2005). Genome-wide strategies for detecting multiple loci that influence complex diseases. Nat. Genet..

[39] Wijayagunawardane MPB, Miyamoto A, Sato K (1999). Prostaglandin E2, prostaglandin F2α and endothelin-1 production by cow oviductal epithelial cell monolayers: effect of progesterone, estradiol 17β, oxytocin and luteinizing hormone. Theriogenology.

[40] Wijayagunawardane MPB (1998). Local distributions of oviductal estradiol, progesterone, prostaglandins, oxytocin and endothelin-1 in the cyclic cow. Theriogenology.

[41] Wijayagunawardane MPB, Kodithuwakku SP, Dé Silva NT, Miyamoto A (2009). Angiotensin II secretion by the bovine oviduct is stimulated by luteinizing hormone and ovarian steroids. J. Reprod. Dev..

[42] Wijayagunawardane MPB, Kodithuwakku SP, Yamamoto D, Miyamoto A (2005). Vascular endothelial growth factor system in the cow oviduct: a possible involvement in the regulation of oviductal motility and embryo transport. Mol. Reprod. Dev..

[43] Parrish JJ, Susko-Panish JL, Winer MA, First NL (1988). Capacitation of bovine sperm by heparin. Biol. Reprod..

[44] Kovacs A, Foote RH (1992). Viability and acrosome staining of bull, boar and rabbit sperm. Biotech. Histochem..

[45] Bianco AM, Martinez E (2009). Robust testing in the logistic regression model. Comput. Stat. Data Anal..

[46] Maalouf M, Trafalis TB (2011). Robust weighted kernel logistic regression in imbalanced and rare events data. Comput. Stat. Data Anal..

[47] MATLAB, version 7.10.0. The MathWorks Inc., Natick, Massachusetts (2010).

[48] Keshtegar B, Allawi MF, Afan HA, El-Shafie A (2016). Optimized river stream-flow forecasting model utilizing high-order response surface method. Water Resour. Manag..

